# The Soup in My Fly: Evolution, Form and Function of Seminal Fluid Proteins

**DOI:** 10.1371/journal.pbio.0060179

**Published:** 2008-07-29

**Authors:** Tracey Chapman

## Abstract

The seminal fluid of males is a complex mixture of biologically potent molecules that shows high evolutionary lability.

The seminal fluid of males from vertebrate and invertebrate taxa is a complex mixture of biologically potent molecules and is far more than a medium to support the successful transit of sperm. But the complexity of this mixture, even in the fly, is only now being fully realised. Recent research is highlighting extraordinarily high evolutionary lability within the genes that encode seminal fluid proteins and is revealing an almost bewildering variety of fitness-related functions. Hence the study of the chemical messages passed from males to females at mating provides a unique window through which to view evolution in action.

It was in the 1960s that details of the nature of the seminal fluid substances that transform the behaviour of female Drosophila melanogaster following mating first started to emerge [1–3]. This elegant work showed that the characteristic refusal of recently mated females to mate again was caused by mating or seminal fluid in the short term and by seminal fluid together with sperm in the longer term [1,2]. The “sex peptide” that was responsible for this effect was identified in 1988 [4]. There then followed first a trickle [5,6] and then an ever increasing stream [7,8] of identifications of the non-sperm seminal fluid proteins [9]. Findlay et al. [10] now provide a tour de force demonstration of the identification of a further 63 proteins, bringing the total so far to 133 proteins confirmed as transferred during mating along with sperm (Figure 1). To put this into perspective, this number represents 35% of the number of proteins (381) found in the Drosophila sperm proteome [11]. What then is the purpose behind such a diverse and biologically active soup?

**Figure 1:**
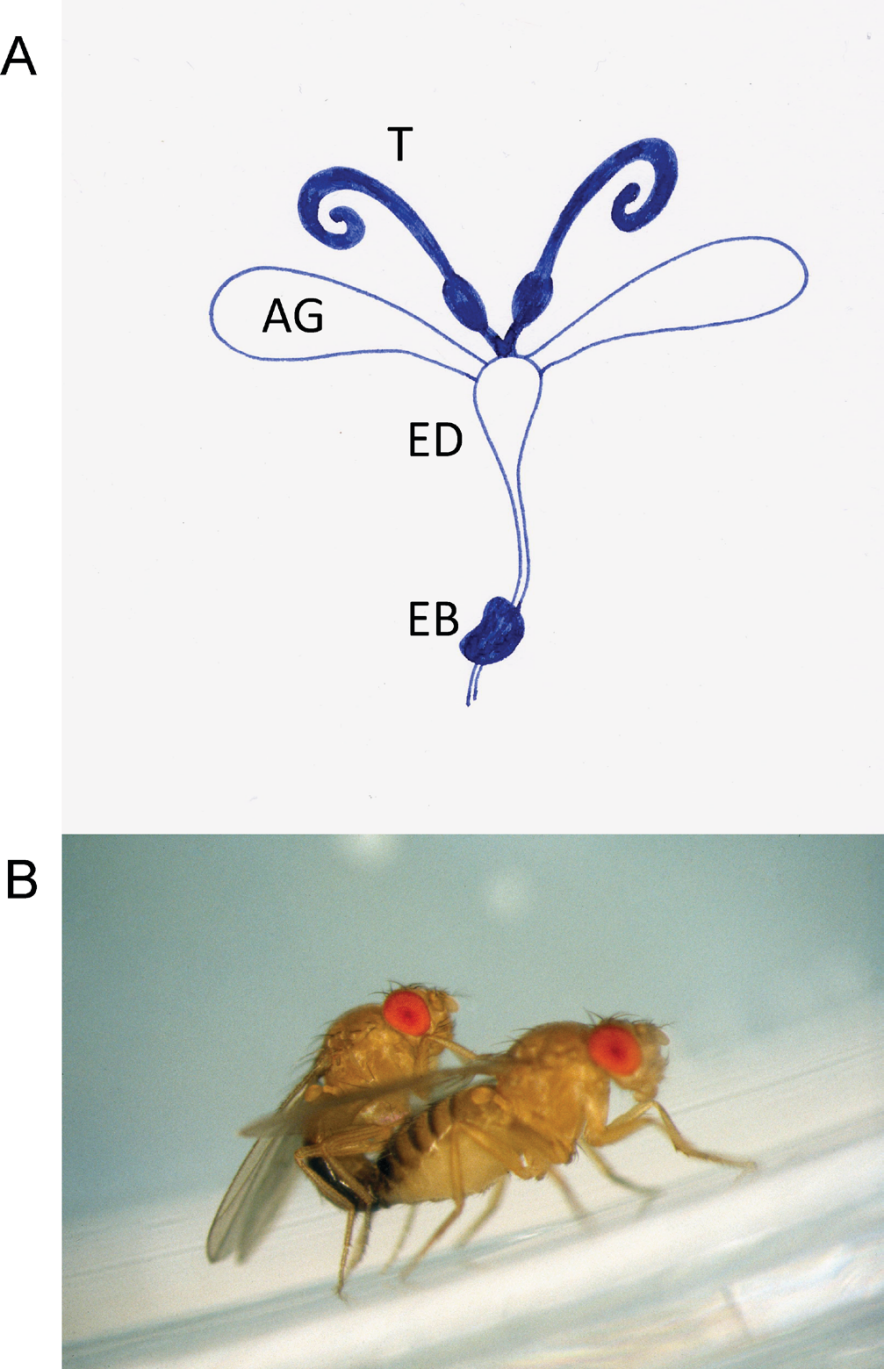
The Soup in My Fly (A) Schematic of the male reproductive system with paired testes (T), paired accessory glands (AG), ejaculatory duct (ED), and ejaculatory bulb (EB). (B) 133 proteins from the accessory glands, ejaculatory duct, and ejaculatory bulb are transferred along with sperm to females during mating and effect profound changes in female behaviour and physiology. (Photo credit: T. Chapman)

## Seminal Fluid Protein Functions and Evolution

Most investigation to date has been done on the largest single class of male seminal fluid proteins, the accessory gland proteins (Acps). Acps are diverse in form, ranging from short peptides to prohormones through to large glycoproteins [12]. Acps cause a wide variety of fitness-related effects, including sperm storage, sperm management and sperm competition, decreased female sexual receptivity, increased egg production (via two different mechanisms), altered morphology of the female reproductive tract, increased production of immune related peptides, increased female feeding, and the liberation of juvenile hormone (JH) (reviewed in [12,13]). Among Acps, there are also proteases, protease inhibitors, lipases, lectins, and cysteine-rich secretory proteins (CRISPs) [14,15]. However, in contrast to the many and varied beneficial functions to males of Acp transfer, the receipt of high levels of Acps can decrease female fitness in a dose-dependent manner [16]. Hence we expect genes that encode seminal fluid proteins such as Acps to be under not only natural and sexual selection, but also to be subject to selection arising from sexual conflict [17]. This combination of strong sexual selection and sexual conflict acting on the evolution of seminal fluid proteins may explain their evolutionary lability (see below).

Recent research is highlighting many intriguing features of seminal fluid protein biology. For example, there is a high level of degeneracy, with each of several functional classes containing multiple proteins [14,15]. There is also evidence of recent Acp gene turnover and recruitment of new Acp genes [18,19]. These observations show that the study of molecular evolution within these genes is likely to shed light on the creation of novel genes and functions and to provide a great opportunity for experimental tests. The fuel for these new insights is coming from ever larger-scale bioinformatic investigations of Acp and seminal fluid gene evolution across different species. An excellent new example comes from the laboratory of Willie Swanson in the new PLoS Biology article by Findlay et al. [10]. The study makes several important contributions and presents technical and bioinformatic advances using genomic data [20]. It presents a fascinating natural history of seminal fluid protein biology, together with deep insight into the mechanisms by which such genes evolve.

Previously, seminal fluid proteins were isolated by purifying substances in male reproductive tracts by high-performance liquid chromatography [4], or by constructing subtracted cDNA libraries of genes expressed in those tissues (e.g., [8]). An evolutionary expressed sequence tag analysis added significantly to the identification of Acps and their rates of evolution by comparing D. melanogaster and D. simulans [9]. However, in all these studies, investigation of whether Acps are actually transferred by males to females has involved predicting which Acps have signal peptides (to indicate which Acps are likely to be secreted) followed by Western blots to check directly for mating transfer (e.g., [21]). Hence previous to this study, only 19 seminal fluid proteins were confirmed as transferred during mating. Findlay et al. have circumvented many of these steps by using proteomic techniques to detect and identify seminal fluid molecules after their transfer to females during mating. They did this by using an ingenious isotopic labelling method, where females were fed heavy nitrogen-labelled yeast, resulting in the labelling of proteins in all female tissues. Mass spectrometry analysis of the reproductive tract proteins isolated from newly mated females could then distinguish between female proteins and those that had been transferred from unlabelled males.

The Findlay et al. study found a total of 133 seminal fluid proteins to be transferred along with sperm during mating, though this may still be an underestimate of the total. This number includes 63 novel and 70 previously predicted seminal fluid proteins and 5 previously described sperm proteins [11]. The transferred seminal fluid proteins fell into unknown, existing, and new functional classes. New members of protease and protease inhibitor classes were found, along with defence/immunity proteins and lipid- and carbohydrate-interacting proteins. The totally new classes identified were odorant binding (7), DNA interacting (3), and chitin-binding (4) proteins. Eleven proteins were identified that were transferred from parts of the male reproductive tract other than the major Acp-producing accessory gland main cells (i.e., the accessory gland secondary cells, the ejaculatory duct, and the ejaculatory bulb); these proteins included esterase-6 [22] and two ejaculatory bulb proteins [23].

The mass spectrometry proteomic techniques used also allowed the relative abundances of the transferred proteins to be determined. This use of this technology opens up the fascinating possibility of testing whether the abundance of seminal fluid proteins transferred is correlated with the magnitude of their phenotype or relationship to fitness. If true, this could reflect the current relative importance of a particular seminal fluid protein in selective terms. Tests for differential allocation of ejaculates by males with different types of females or by males held under different evolutionary regimes are also an exciting possibility. Findlay et al also used new bioinformatic methods to identify novel and previously unknown, unannotated genes. This was done by making predicted translations of the euchromatic genome, which resulted in the identification of 19 novel seminal fluid proteins with signal sequences that had been overlooked in all previous genome annotations. One wonders how many more Drosophila genes have been similarly overlooked.

It has previously been observed that some seminal fluid protein genes evolve very rapidly [9,18,19,24–26]. The Findlay et al. study takes this further, with a comprehensive set of tests for positive selection in 36 of the transferred seminal fluid proteins, finding evidence for positive selection in 16 of these and narrowing down the specific residues under selection, by using the powerful methods developed by Ziheng Yang and Rasmus Nielsen [27]. As more species comparisons are added to such analyses, the increased statistical power gained is likely to increase still further the number of positively selected Acps that are detected. An additional feature of the evolution of seminal fluid protein genes is the emergence of gene clusters. Nineteen clusters of 2–5 seminal fluid protein genes were found, of which 15 contained genes with full-length homology to one another, suggesting origin by tandem duplication. This suggests that duplication in clusters may allow new functions to be adopted by one or more of the duplicated genes. This, together with exciting work that shows evidence for evolutionarily rapid gene loss and gain [18,19], suggests that among genes that encode seminal fluid proteins, there is striking evolutionary lability in not only rapid selection of specific residues, but in gene rearrangements and even gene creation and loss. One consequence of rapid gene turnover is that some seminal fluid encoding genes are likely to be lineage or species specific. Analysis of the 12 Drosophila species genomes shows that many of the 44 lineage-specific genes identified are in fact male reproductive proteins including Acps [20]. Of the seminal fluid proteins studied by Findlay et al. in D. melanogaster, D. simulans, and D. yakuba, 13 were found to be lineage specific, including at least one example of a gene that appeared to have lost sex-limited expression in D. melanogaster.

## Why so Many Seminal Fluid Proteins?

Surely 133 proteins to transfer along with sperm is an embarrassment of riches? To date, seminal fluid proteins are unevenly divided between just 12 functional classes, but with the majority being in the protease, protease inhibitor, immunity, and lipid metabolism categories. It is an intriguing and unanswered question of seminal fluid biology as to why there are multiple representatives of each type. It will be interesting to test whether each seminal fluid protein produces a distinct phenotype (Table 1). One explanation for the apparent abundant degeneracy is that the male reproductive system carries the baggage of previous evolutionary contests between males or between males and females. Hence one member of each functional class is the current “favourite”, but declines in importance when sexual competition becomes too intense, or when one side becomes insensitive to the actions of the current player (Table 1). A contrasting situation is apparently found for one of the best functionally characterised seminal fluid proteins, the sex peptide (SP). SP is only 36 amino acids long, but affects at least five distinct phenotypes (egg production, female receptivity, increased female feeding, antimicrobial peptide production, and JH synthesis from corpora allata) that are encoded by at least two different functional domains (reviewed in [28]). There are clearly important roles for many other seminal fluid proteins (e.g., [13]), but why some molecules such as SP appear to have monopolised more than their fair share of functions is not yet clear.

**Table 1:**
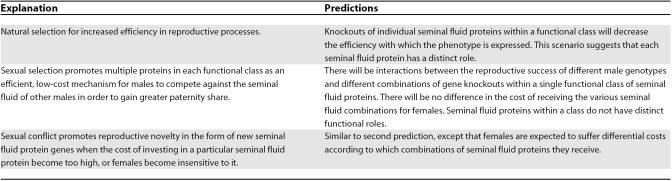
Possible Explanations for Degeneracy in Seminal Fluid Proteins

## New Classes of Seminal Fluid Proteins

Another interesting feature highlighted by the new research is that yet more new classes of seminal fluid proteins are being identified. For example, Findlay et al. show that some odorant-binding proteins (Obps) are previously unknown seminal fluid proteins that are transferred in abundance. Obps [29] transport odorant molecules to their receptors and may play a role in pheromone communication during sex. The abundance of Obps can differ between males and females [e.g., 30], and one Obp99b is reported to be up-regulated in courting males [31]. Transcriptional changes in Obps are also reported from lines selected for fast and slow mating latency [32], with ten Obps showing altered expression in both sexes or in males only. The FlyAtlas database [33] shows that of the 51 Obp-coding genes currently known in D. melanogaster, eight appear specific to the testis and/or accessory glands. However, it was not known until this present study that some Obps are transferred to females during mating. Some Obp genes occur as clusters within the genome, and in these clusters, there are Obps whose expression is restricted to either the head, head and male reproductive system, or reproductive system (data from [33]). This pattern is shown in the nine genes of the Obp56 gene cluster, four members of which are now confirmed to be transferred seminal fluid proteins [10]. Using such clusters, it will be possible to test the hypothesis that duplications of genes in clusters followed by the evolution of tissue specificity allows genes that take on reproductive functions to evolve more rapidly than their ancestral counterparts. If the role of Obps in the reproductive system is similar to that of Obps in the antennae, then perhaps they facilitate chemotaxis of sperm to the egg [34,35]. Such a hypothesis predicts that organisms lacking Obps in the reproductive system will show fertility defects.

## Distribution of Seminal Fluid Protein Genes

The newest study again confirms the statistically significant lack of seminal fluid protein gene representation on the X chromosome [10]. Seminal fluid proteins are predicted to be subjected to sexually antagonistic coevolution because their effects can benefit males but cause costs in females [16]. However, the X chromosome is also predicted to be a hot spot for sexually antagonistic genetic variation [36]. How are these findings to be reconciled? One possibility is that dominance relationships may be important, as genes with dominant effects that favour male fitness above that of females would be expected to be less favoured on the X [37]. It will therefore be interesting to compare the functions of the few X-linked with the majority of non–X-linked seminal fluid protein genes.

To conclude, the availability of genome sequences together with recent advances in proteomics and in analysis of the identification, characterisation, and molecular evolution of male reproductive genes are opening up exciting new avenues for research and are revealing the extraordinary lability in reproductive genes.
